# A Treatise on Diagnostic Methods of Examination

**Published:** 1905-12

**Authors:** 


### BOOK REVIEWS.

A Treatise on Diagnostic Methods of Examination. By Prof. Dr. H. Sahli, of Bern. Edited, with additions, by Francis P. Kinnicutt, M.D., Professor of Clinical Medicine, Columbia University, N. Y.; and Nath'l Bowditch Potter, M.D., Visiting Physician to the City Hospital and to the French Hospital; and Consulting Physician to the Manhattan State Hospital, N. Y. Philadelphia and London: W. B. Saunders & Company, 1905. Octavo of 1008 pages, profusely illustrated. Cloth, $6.50 net; Half Morocco $7.50 net.

We have been anxiously awaiting the publication of Dr. Sahli's great work in English. Its immediate success in Germany will certainly be repeated in this country, and the English-speaking profession owe to Messrs. W. B. Saunders & Company a debt of gratitude for their enterprise. Not only does the distinguished professor exhaustively consider all methods of examination for the purpose of diagnosis, but the explanations of clinical phenomena are given and discussed from physiologic as well as pathologic points of view, and with a thoroughness never before attempted in any clinical work. The examinations of the stomach, sputum, feces, urine, and blood are exhaustively treated. There is an article from the pen of Dr. Theodore C. Janeway giving a brief review of the investigations of American and English observers upon the value of the clinical estimation of blood-pressure, with a description of some newly devised instruments. Some of the new features in the chapter on urine examination are: Seliwanow's reaction for levulose, Bial's test for pentoses, and quantitative determination of urochrome after Klemperer. Osmotic pressure and cryoscopy of the urine are also discussed at length, and a description is given of Liebermann and Posner's method of staining urinary pigments. In the chemical examination much attention is directed to describing methods; and this is done so exactly that it is possible for the clinician to work according to these directions. The nervous system has been very elaborately detailed, giving unusual space to electrical examination. Indeed, the American edition of this great work contains all the material of the new fourth German edition, with which it simultaneously appeared. Many new illustrations have been added by the editors. The work is indispensable to the practitioner.

